# Supplementary material for: AuPairWise: A Method to Estimate RNA-Seq Replicability through Co-expression
Source: PLoS Comput Biol. 2016 Apr 15;12(4):e1004868. doi: 10.1371/journal.pcbi.1004868 (PMC4833304; doi:10.1371/journal.pcbi.1004868)
Supplement: S1 Text — (DOCX) [file pcbi.1004868.s001.docx]

# Supplementary Material

# AuPairWise: a method to estimate RNA-seq replicability through co-expression

### Sara Ballouz, Jesse Gillis*

Stanley Institute for Cognitive Genomics, Cold Spring Harbor Laboratory, Cold Spring Harbor, NY, USA

*Corresponding author

Email addresses:

SB: [sballouz@cshl.edu](mailto:sballouz@cshl.edu)

JG: [jgillis@cshl.edu](mailto:jgillis@cshl.edu)

## Table of Contents

[Supplementary Methods 2](#_Toc441612535)

[Effects of normalization 3](#_Toc441612536)

[Supplementary Figures 4](#_Toc441612537)

[Supplementary Tables 4](#_Toc441612538)

[Supplementary data 13](#_Toc441612539)

[Data: 13](#_Toc441612540)

[Code: 13](#_Toc441612541)

[References 14](#_Toc441612542)

## Table of Supplementary Figures

[Fig. S1 Density of mean gene-gene correlations between replicates before and after filtering. 4](#_Toc441613057)

[Fig. S2 AuPairWise noise metrics on RNA-seq experiments. 5](#_Toc441613058)

[Fig. S3 Average AUROC using alternative gene-pairs and different expression normalization methods. 6](#_Toc441613059)

[Fig. S4 Distribution of the gene-pair AUROCs for different noise factor parameters. 7](#_Toc441613060)

[Fig. S5 Variance versus parameter choices in the AuPairWise method. 8](#_Toc441613061)

[Fig. S6 Performance AUROCs for AuPairWise on ENCODE data with the 2-fold cross validation. 8](#_Toc441613062)

[Fig. S7 Performance AUROCs for AuPairWise on microarray and RNA-seq versions of the BrainSpan dataset. 9](#_Toc441613063)

[Fig. S8 Expansion of Fig6C explaining the noise model. 10](#_Toc441613064)

## Table of Supplementary Tables

[Table S1 GO enrichment of genes in the housekeeping interaction gene pairs 11](#_Toc441613295)

[Table S2 Significance of difference in performance AUROCs for gene-pairs compared to random pairs of the ENCODE dataset. 11](#_Toc441613296)

[Table S3 Significance of difference in performance AUROCs for gene-pairs compared to random pairs for microarray and RNA-seq versions of the Brainspan dataset. 11](#_Toc441613297)

[Table S4 Performance and significance of difference in performance AUROCs for gene-pairs compared to random pairs for 2-fold cross validation of the ENCODE dataset. 12](#_Toc441613298)

[Table S5 Average running time of AuPairWise across different sample sizes and repeats for a noise factor 12](#_Toc441613299)

**Supplementary Text**

This supplement contains additional figures and tables referenced in the main text, along with some additional text on normalization issues. In **S1 Fig**, we show that the correlation threshold used for a reproducible gene, although arbitrary, is similar to using the mean correlation. **S2 Fig** shows the results of AuPairWise on a collection of 83 RNA-seq experiments. **S3 Fig, S5 Fig, S6 Fig** and **S7** **Fig** are the response curves for varying parameters of AuPairWise. We show the distributions of the AUROCs for random and stoichiometric pairs in **S4 Fig**. And finally, we expand on the perturbation model in **S8 Fig**.

**S1** **Table** lists the GO terms enriched in the stoichiometric pairs. **S2** **Table** and **S3** **Table** list the average AUROCs and the corresponding p-values for the ENCODE and BrainSpan experiments. **S4** **Table** corresponds to the values in **S6 Fig**. We evaluate the run times for different sized experiments in **S5** **Table**.

## Supplementary Methods

### Effects of no**r**malization

To check if normalization affected the performance of AuPairWise, we ran our method on our reference dataset after it had been normalized using some popular methods. We took the ENCODE raw data from SRA and ran it through a standard RNA-seq pipeline (bowtie2 [[1](#_ENREF_1)]+ HTseq[[2](#_ENREF_2)]) to get count data. We generated RPKM values from bowtie2 + RSEQtools[[3](#_ENREF_3)]. The count data was normalized through the trimmed mean of m-values (TMM) method from edgeR[[4](#_ENREF_4)] and the variance stabilizing transformation (VST) method from DESeq[[5](#_ENREF_5)]. We normalized the RPKM data to TPM by standardizing each sample by its total read depth. As a final comparison, we ranked the RPKM values (by sample), and used that as another normalization method. All 5 versions of this data set (except the raw counts), were run through AuPairWise across 14 noise factors with a 1000 repeats each. Individually, the co-expressed pairs and the random pairs were used to calculate the performance (average AUROC per noise factor across all the repeats/runs). See **S3** **Fig**.

## Supplementary Figures

**
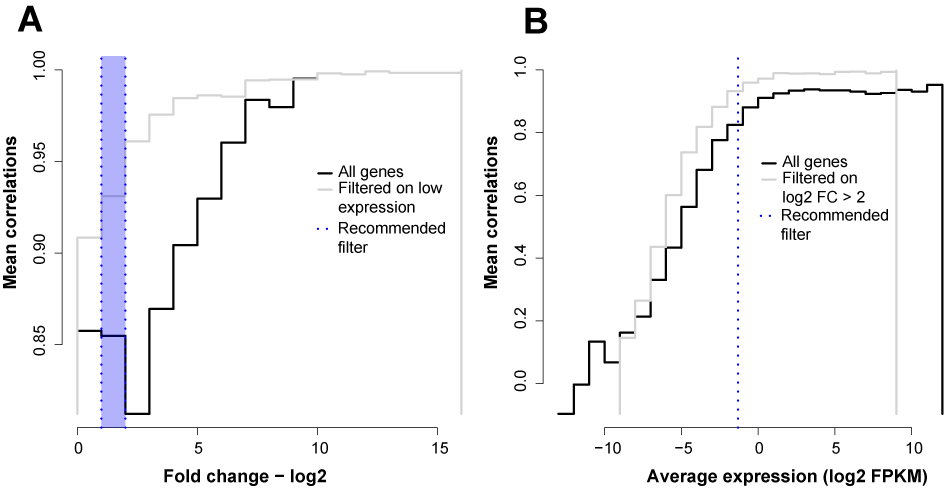
**

S1 Fig Density of mean gene-gene correlations between replicates before and after filtering.

(A) For the fold change values, we see that for the recommended FC cutoffs (the blue dotted lines), the mean correlations are low and increase the further we move (black line). Removing low expressing genes shows even higher mean correlations (grey line). (B) For the average expression values, once again, we see that for the recommended cut-off, we have higher mean correlations (black line), that are improved even more with the inclusion of the fold change filter (grey line).


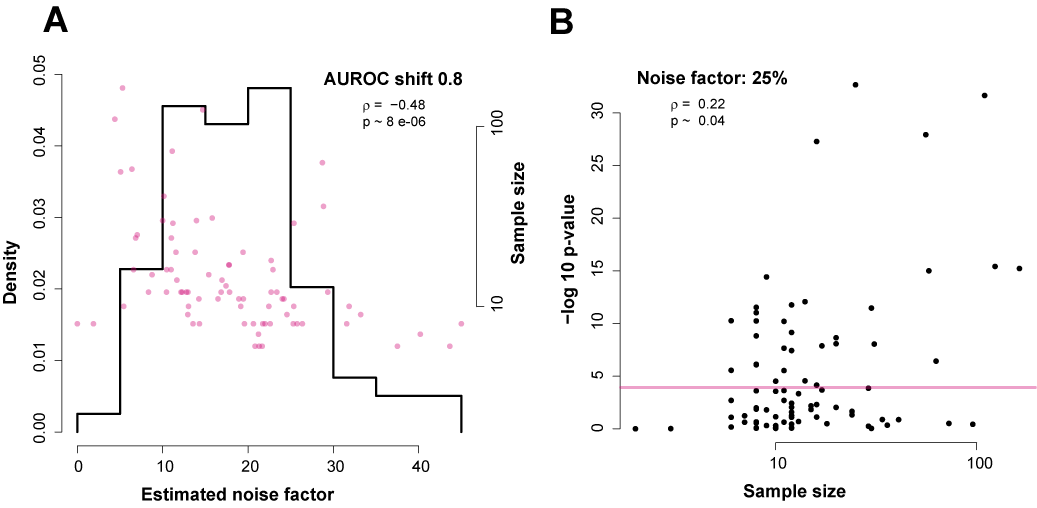


**S2 Fig** **AuPairWise noise metrics on RNA-seq experiments.**

Here we took 83 RNA-seq experiments and ran the AuPairWise method for 100 repeats across a range of noise factors. (A) The density plot (solid line) shows that most experiments fall within the 19% noise factor range as assessed to cause an AUROC shift to 0.8. There is a dependence on sample size of course, with larger experiments requiring smaller noise perturbations (data points in figure). (B) Significance of difference in performance AUROCs for biologically related gene-pairs compared to random pairs for all the experiments. The geometric mean of these p-values is 4.67e-06. For comparison, the ENCODE reference dataset with 18 samples sits with a p-value of 6.52-e14.


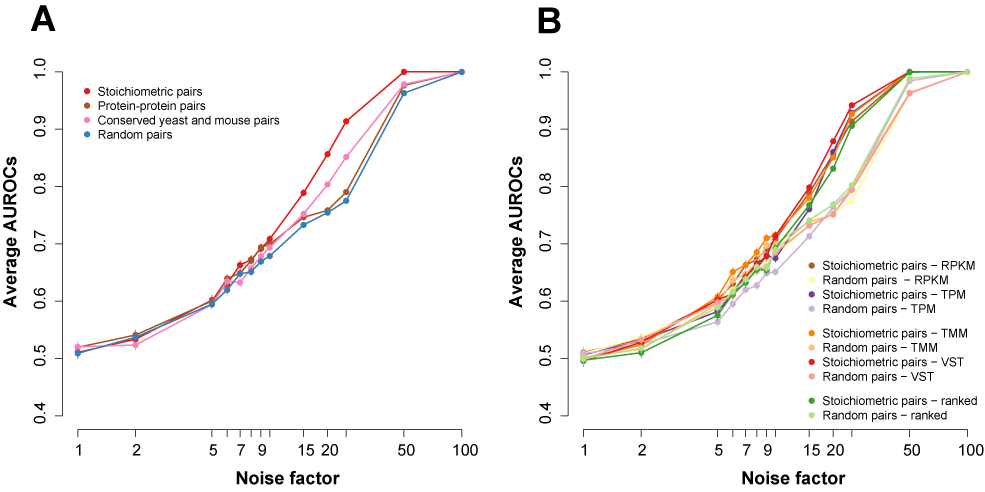


**S3 Fig Average AUROC using alternative gene-pairs and different expression normalization methods.**

(A) The co-expressed pairs (shown in red) outperform the protein-protein pairs (brown), the conserved yeast-mouse co-expressed pairs (pink) and random pairs (blue). These are the results of 1000 repeats. (B) Using different normalization methods on the expression data has a small effect on the performance AUROCs of the stoichiometric pairs. Here we compared the effects of RPKM (brown), TPM (blue), TMM (orange), VST (red), and simply ranking the expression data (green). These are the results of 1000 repeats.


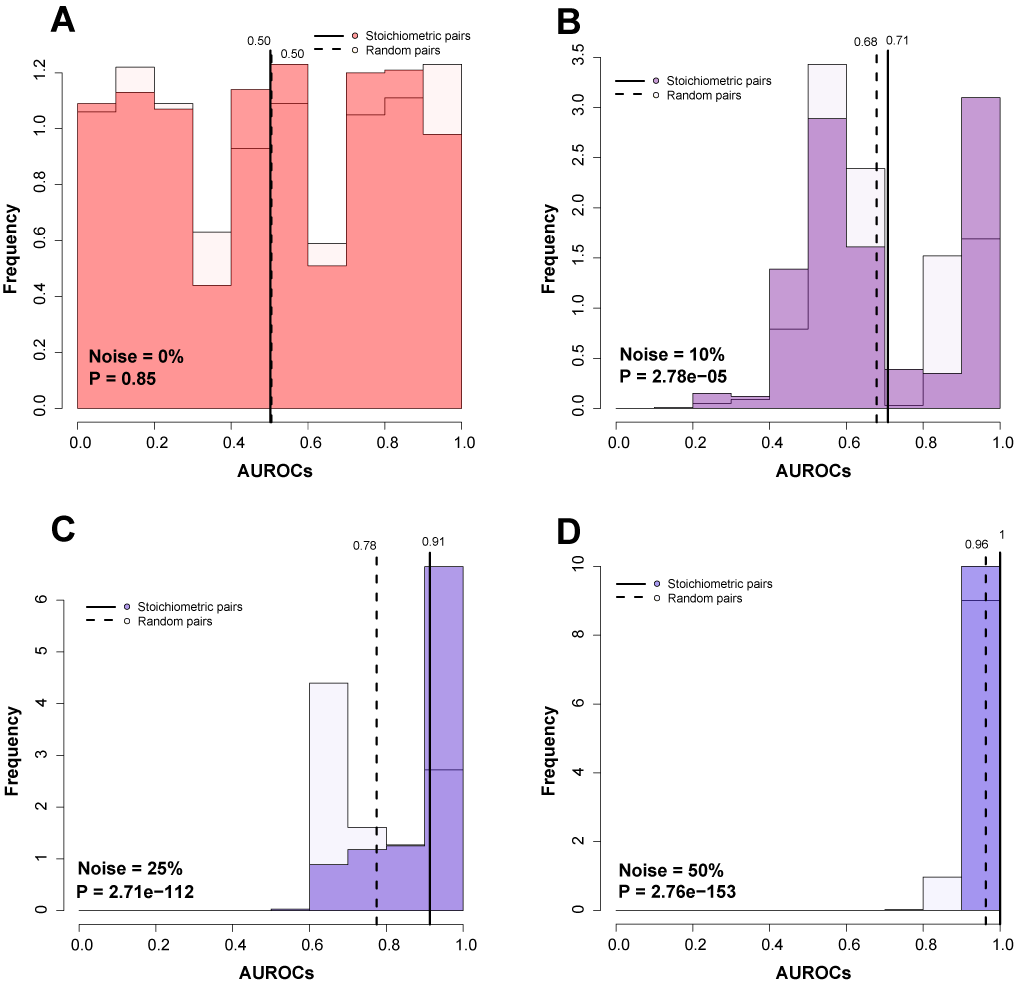


**S4 Fig** **Distribution of the gene-pair AUROCs for different noise factor parameters.**

The distributions are for 1000 runs of the method on the same dataset, varying the sample being perturbed. The colored distribution is the co-expressed pair performances, while the lighter distribution is the performance of an equal number of random gene-pairs. The solid line is the average AUROC for the co-expressed pairs, the dashed for the random pairs. The p-value is the significance of the difference between these two distributions (Wilcoxon test, two-sided). Panels are for different noise factors (A) No noise added (p~0.85), (B) 10% noise (p~2.78e-5), (C) 25% (p~2.71e-112) and (D) 50% (p~2.76e-153). We see that although the average AUROCs may be similar, the distributions are significantly different.


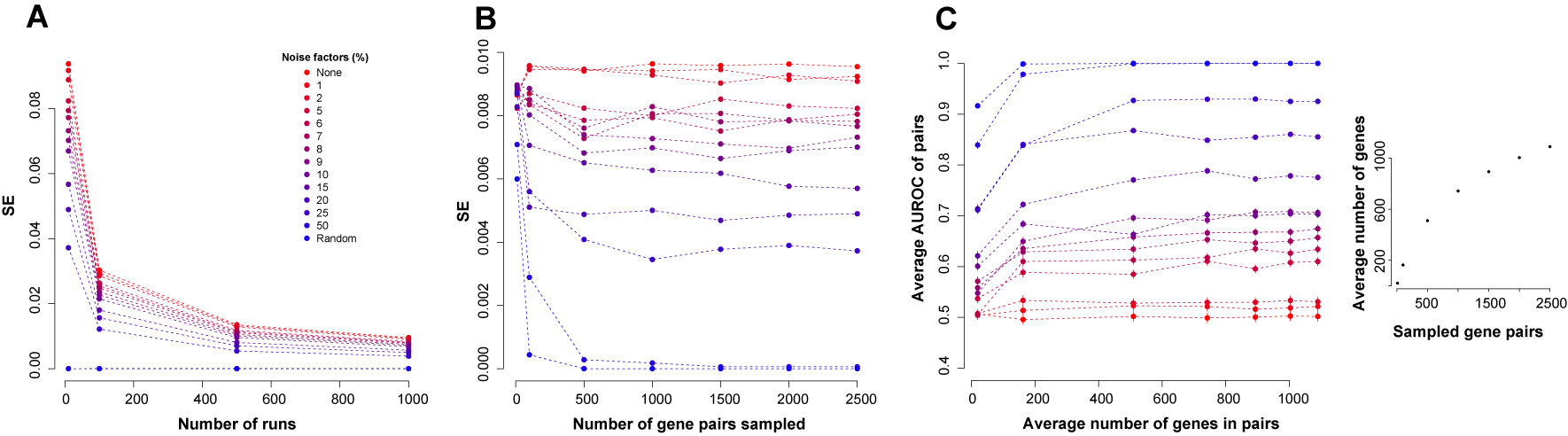


**S5 Fig** **Variance versus parameter choices in the AuPairWise method.**

Here we show the standard error (SE) of the performance AUROCs of the stoichiometric pairs as a function of the parameter choices on the ENCODE dataset, holding the sample size constant. (A) First we calculate the effect of the number of runs on the analysis, by varying the number of repeats from 10 to 1000. The SE decreases as the number of runs increases, as expected, with the noise factor having a small effect on the SE. (B) Varying gene-pair numbers (between 10 and 2500) shows a consistent SE only when 500 or more gene-pairs are used. To note, all variance is below 0.01 for almost all these parameter choices, indicating the results are quite robust. (C) We show the performances (average AUROCS) as a function of to the average number of genes within the sampled gene-pairs (and mapping between average number of genes and gene-pairs in inset).


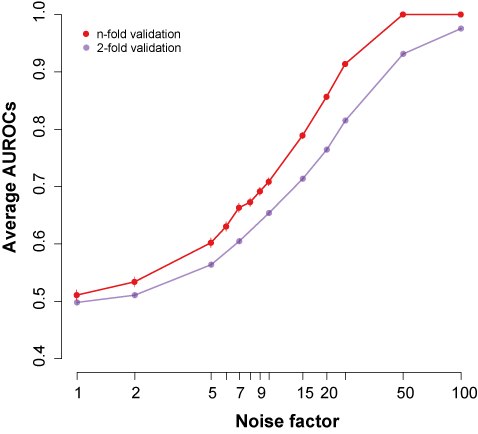


**S6 Fig** **Performance AUROCs for AuPairWise on ENCODE data with the 2-fold cross validation.**

Using the ENCODE dataset once again and running the method 1000 times, we varied the number of samples perturbed in the experiment. The n-fold cross-validation (one perturbed sample, in red) outperform 2-fold cross-validation (half the samples perturbed, purple). The 2-fold validation does not have an AUROC performance of 1 at 100% (~0.98), likely due to the fact that a great deal of co-expression is lost, hence preventing the method from detecting any structured co-variation.


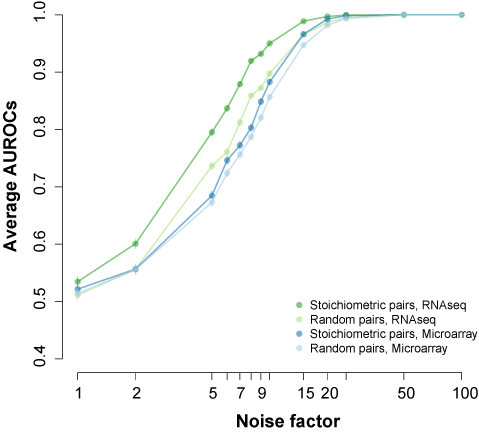


**S7 Fig** **Performance AUROCs for AuPairWise on microarray and RNA-seq versions of the BrainSpan dataset.**

The co-expressed pairs (darker lines) outperform the random pairs (lighter lines), for both the RNA-seq (green) and Microarray (blue) datasets. But the microarray dataset has more similar performances between the co-expressed pairs and the random pairs, compared to the RNA-seq version. The microarray dataset also performs worse/close to the RNA-seq random pairs.


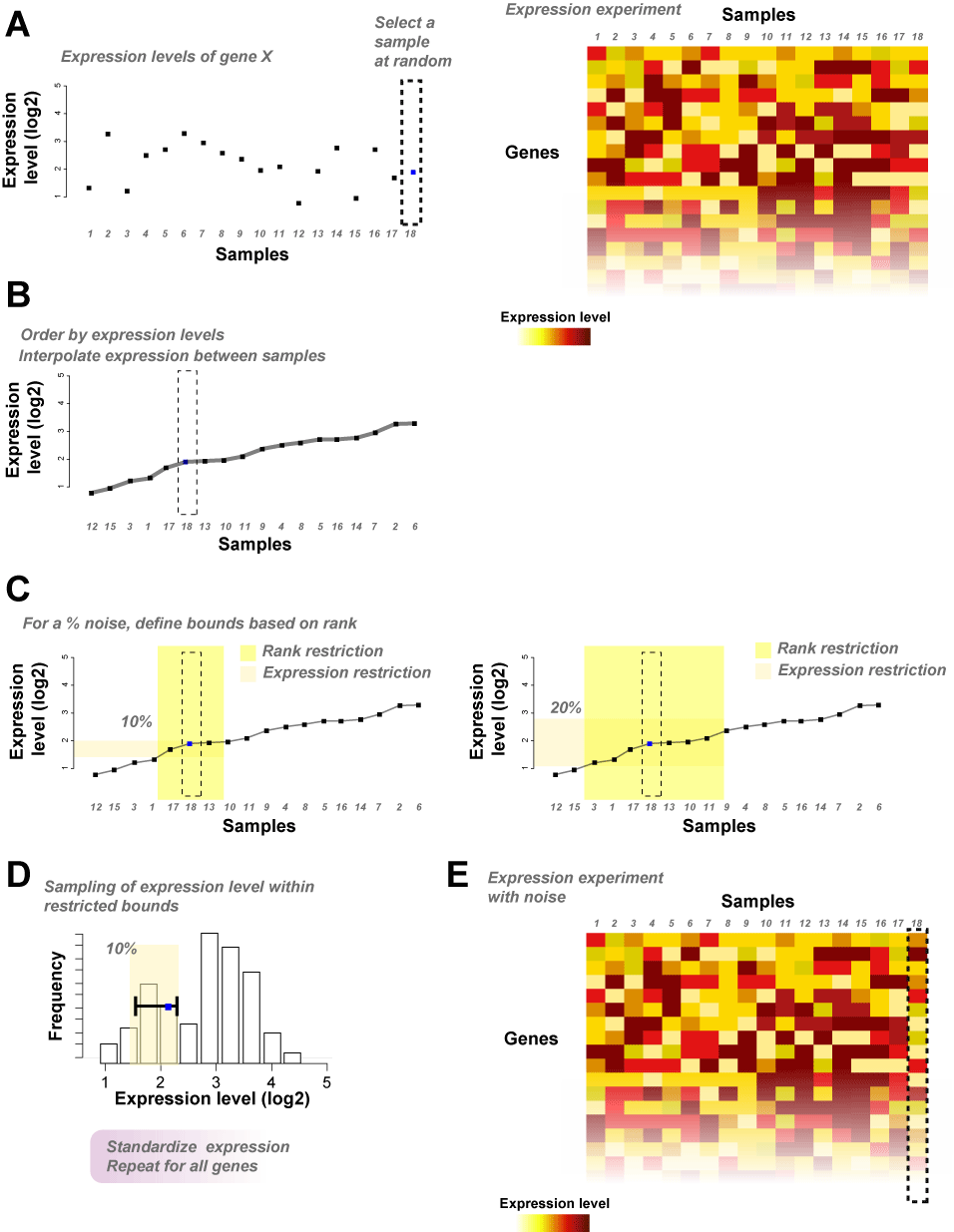


**S8 Fig** **Expansion of Fig6C explaining the noise model.**

(A) Expression of gene X from experiment. (B) The first step is to order the expression levels, and interpolate between the samples to get a continuous distribution of expression. (C) Then with this distribution, we define the boundaries for the noise included expression levels for that gene. For instance, with 10% noise, the new rank is limited to the yellow rectangle bounds, which corresponds to an expression change, constrained between expression values of ~1 to ~2 (the orange rectangle). (D) We then pick the new expression level and standardize it. (E) This is repeated until all the genes in the selected sample have “noise” added to them.

## Supplementary Tables

S1 Table GO enrichment of genes in the housekeeping interaction gene pairs

**S2 Table** **Significance of difference in performance AUROCs for gene-pairs compared to random pairs of the ENCODE dataset.**

**S3 Table** **Significance of difference in performance AUROCs for gene-pairs compared to random pairs for microarray and RNA-seq versions of the Brainspan dataset.**

**S4 Table** **Performance and significance of difference in performance AUROCs for gene-pairs compared to random pairs for 2-fold cross validation of the ENCODE dataset.**

**S5 Table** **Average** **running time of AuPairWise across different sample sizes and repeats for a noise factor**

## Supplementary data

All code and data is located in: github.com/sarbal/AuPairWise

We briefly describe what is available here.

### Data:

Located in the *sample* directory:

ENCODE: **sample_ENCODE.Rdata**

BrainSpan: **sample_brainspan.Rdata**

Located in the *suppl* directory:

Liver and kidney expression datasets: **corrs-examples.Rdata**

### Code:

Located in the *bin* directory:

AuPairWise code and helper scripts:

**AuPairWise.r**

**helper.R**

Scripts to run AuPairWise that can be copied or modified:

**run_AuPairWise_on_data.r**

**run_ on_sample_data.r**

Located in the *suppl* directory:

Code to perform analyses

**AuPairWise_analysis.r**

## References

1. Langmead B, Salzberg SL (2012) Fast gapped-read alignment with Bowtie 2. Nature Methods 9: 357-359.

2. Planet E, Stephan-Otto Attolini C, Reina O, Flores O, Rossell D (2011) htSeqTools: High-Throughput Sequencing Quality Control, Processing and Visualization in R. Bioinformatics.

3. Habegger L, Sboner A, Gianoulis TA, Rozowsky J, Agarwal A, et al. (2011) RSEQtools: a modular framework to analyze RNA-Seq data using compact, anonymized data summaries. Bioinformatics 27: 281-283.

4. Robinson MD, McCarthy DJ, Smyth GK (2010) edgeR: a Bioconductor package for differential expression analysis of digital gene expression data. Bioinformatics 26: 139-140.

5. Anders S, Huber W (2010) Differential expression analysis for sequence count data. Genome Biol 11: R106.
