# Supplementary figures and images for: AuPairWise: A Method to Estimate RNA-Seq Replicability through Co-expression
Source: PLoS Comput Biol. 2016 Apr 15;12(4):e1004868. doi: 10.1371/journal.pcbi.1004868 (PMC4833304; doi:10.1371/journal.pcbi.1004868)

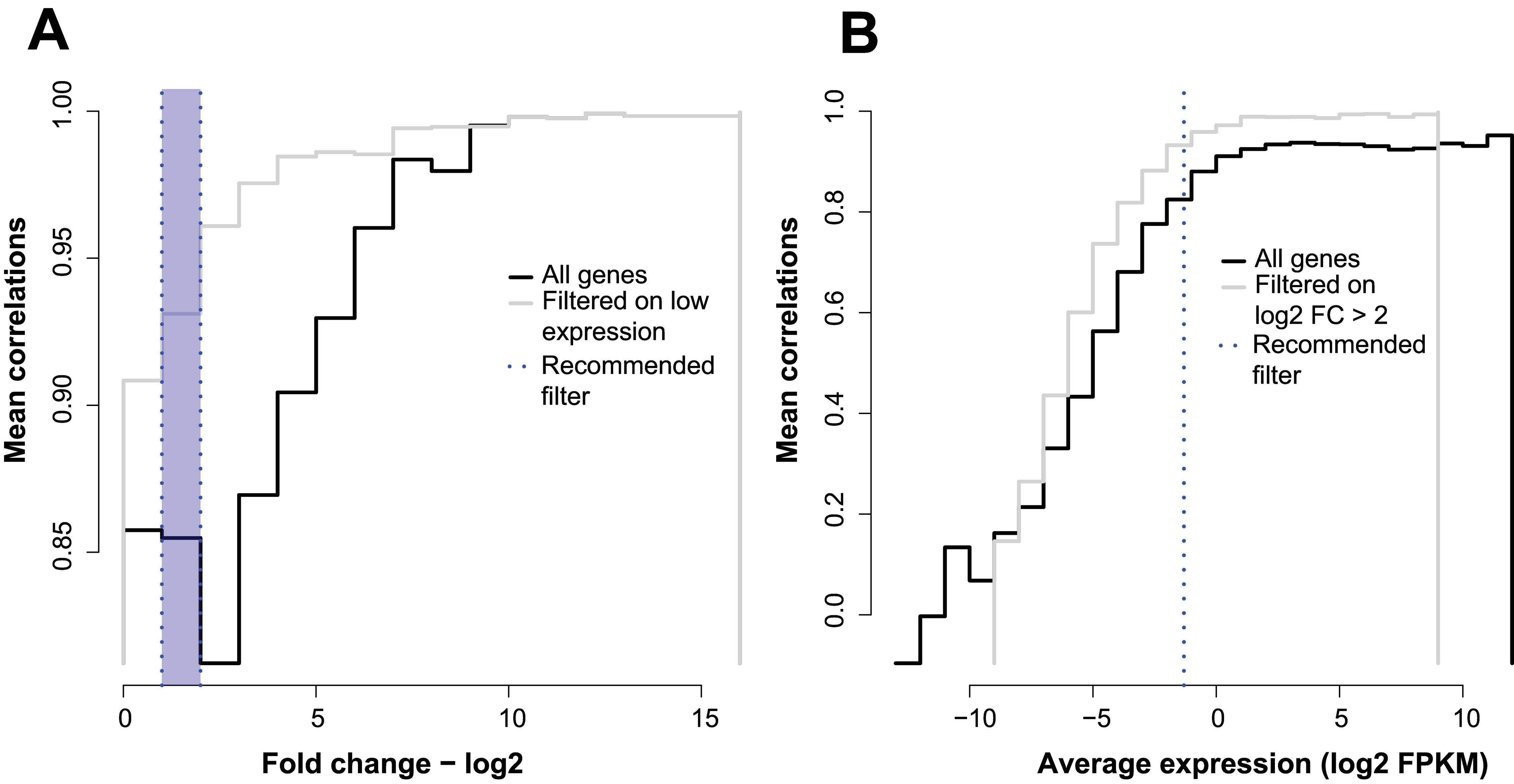

Supplement: S1 Fig — (A) For the fold change values, we see that for the recommended FC cutoffs (the blue dotted lines), the mean correlations are low and increase the further we move (black line). Removing low expressing genes shows even higher mean correlations (grey line). (B) For the average expression values, once again, we see that for the recommended cut-off, we have higher mean correlations (black line), that are improved even more with the inclusion of the fold change filter (grey line). (TIF) [file pcbi.1004868.s002.tif]

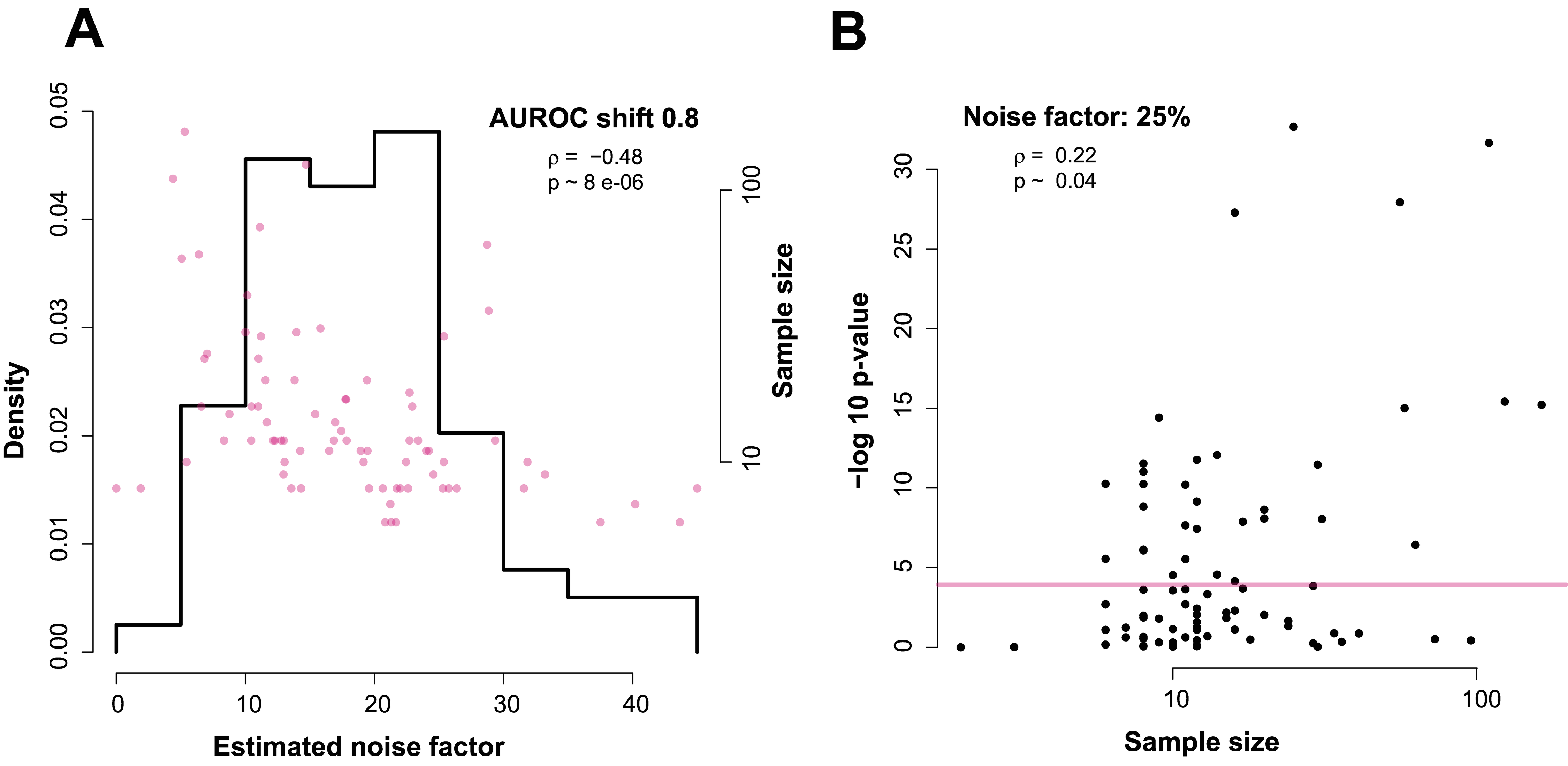

Supplement: S2 Fig — Here we took 83 RNA-seq experiments and ran the AuPairWise method for 100 repeats across a range of noise factors. (A) The density plot (solid line) shows that most experiments fall within the 19% noise factor range as assessed to cause an AUROC shift to 0.8. There is a dependence on sample size of course, with larger experiments requiring smaller noise perturbations (data points in figure). (B) Significance of difference in performance AUROCs for biologically related gene-pairs compared to random pairs for all the experiments. The geometric mean of these p-values is 4.67e-06. For comparison, the ENCODE reference dataset with 18 samples sits with a p-value of 6.52-e14. (TIF) [file pcbi.1004868.s003.tif]

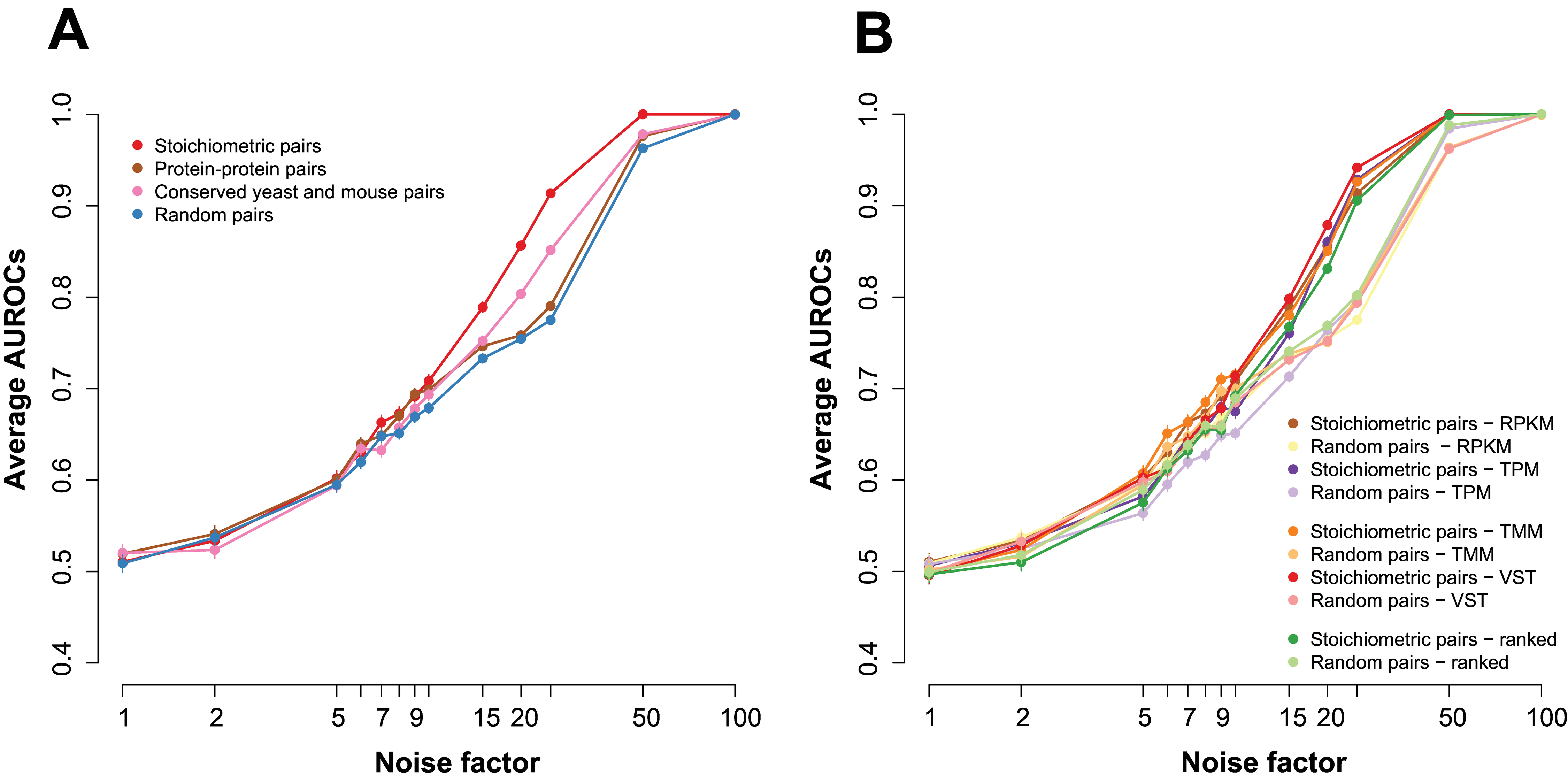

Supplement: S3 Fig — (A) The co-expressed pairs (shown in red) outperform the protein-protein pairs (brown), the conserved yeast-mouse co-expressed pairs (pink) and random pairs (blue). These are the results of 1000 repeats. (B) Using different normalization methods on the expression data has a small effect on the performance AUROCs of the stoichiometric pairs. Here we compared the effects of RPKM (brown), TPM (blue), TMM (orange), VST (red), and simply ranking the expression data (green). These are the results of 1000 repeats. (TIF) [file pcbi.1004868.s004.tif]

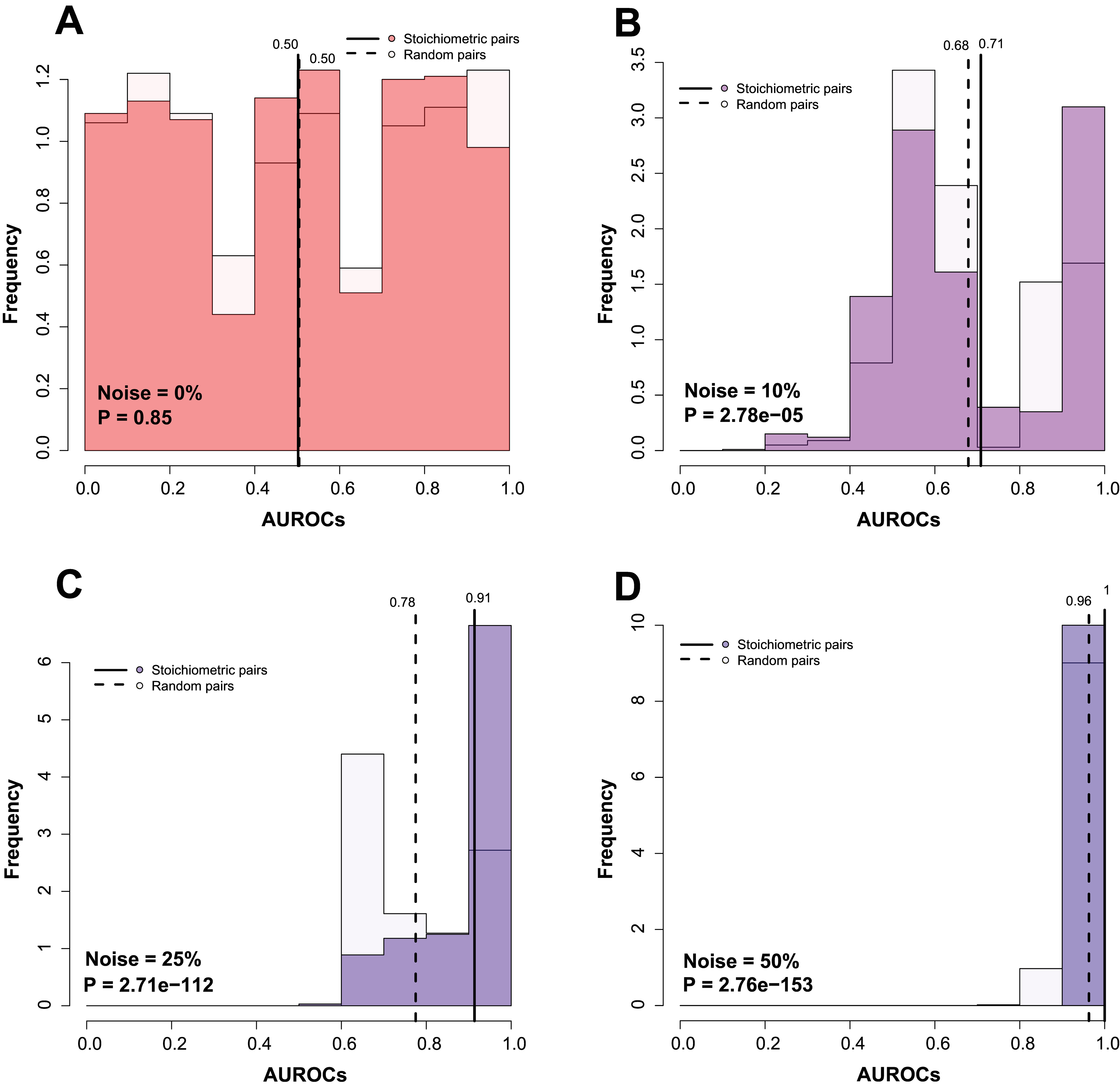

Supplement: S4 Fig — The distributions are for 1000 runs of the method on the same dataset, varying the sample being perturbed. The colored distribution is the co-expressed pair performances, while the lighter distribution is the performance of an equal number of random gene-pairs. The solid line is the average AUROC for the co-expressed pairs, the dashed for the random pairs. The p-value is the significance of the difference between these two distributions (Wilcoxon test, two-sided). Panels are for different noise factors (A) No noise added (p~0.85), (B) 10% noise (p~2.78e-5), (C) 25% (p~2.71e-112) and (D) 50% (p~2.76e-153). We see that although the average AUROCs may be similar, the distributions are significantly different. (TIF) [file pcbi.1004868.s005.tif]

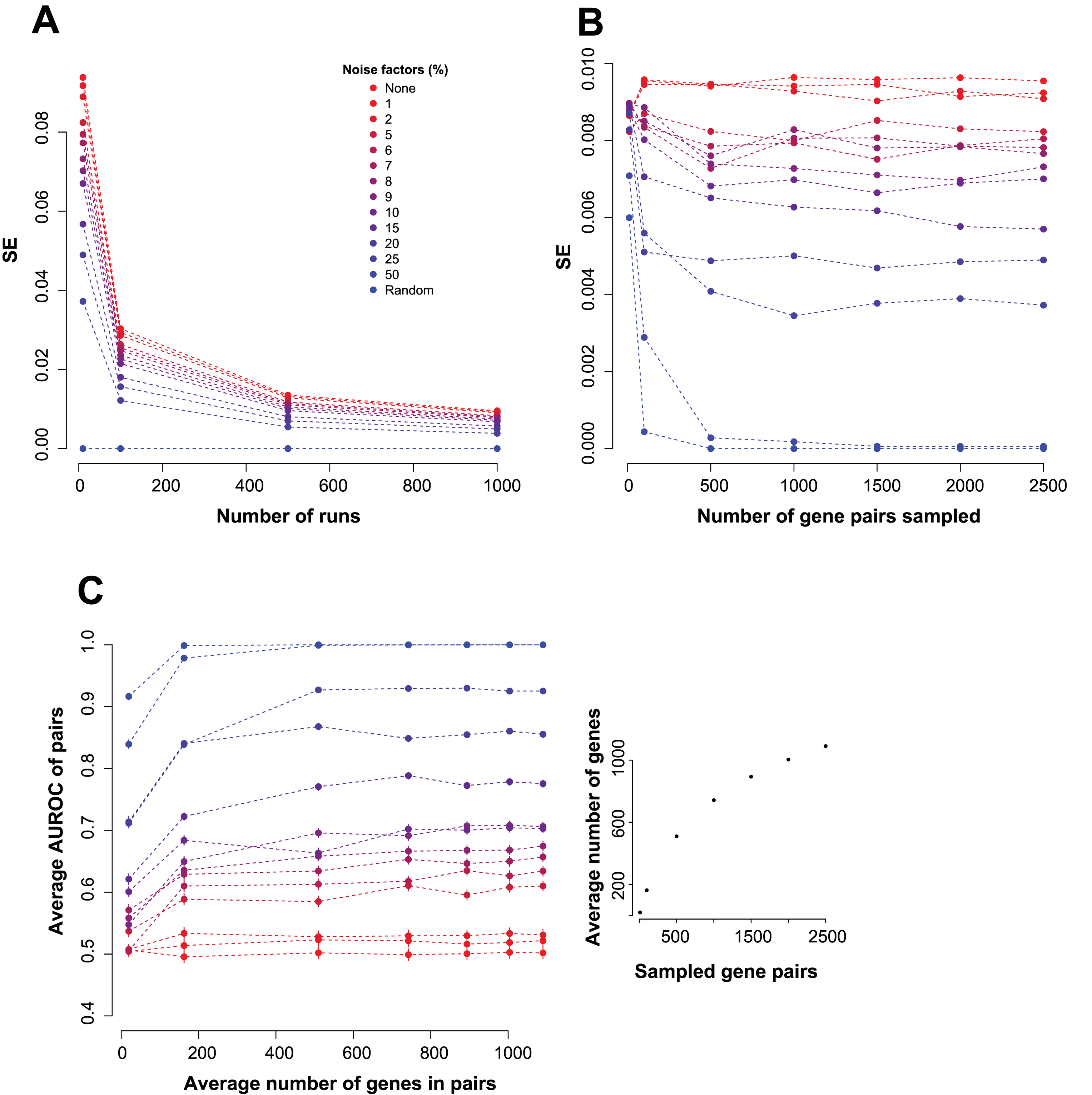

Supplement: S5 Fig — Here we show the standard error (SE) of the performance AUROCs of the stoichiometric pairs as a function of the parameter choices on the ENCODE dataset, holding the sample size constant. (A) First we calculate the effect of the number of runs on the analysis, by varying the number of repeats from 10 to 1000. The SE decreases as the number of runs increases, as expected, with the noise factor having a small effect on the SE. (B) Varying gene-pair numbers (between 10 and 2500) shows a consistent SE only when 500 or more gene-pairs are used. To note, all variance is below 0.01 for almost all these parameter choices, indicating the results are quite robust. (C) We show the performances (average AUROCS) as a function of to the average number of genes within the sampled gene-pairs (and mapping between average number of genes and gene-pairs in inset). (TIF) [file pcbi.1004868.s006.tif]

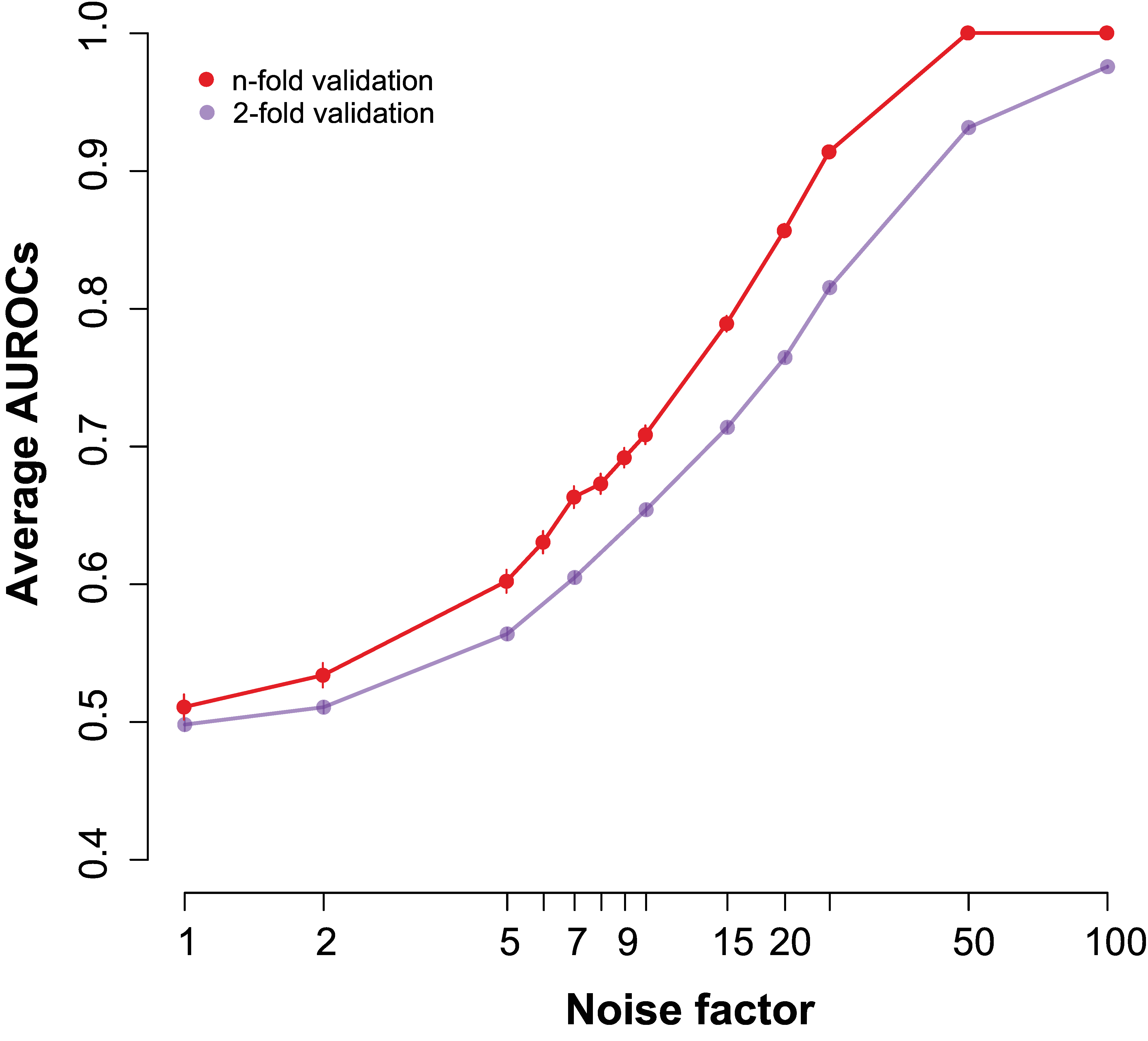

Supplement: S6 Fig — Using the ENCODE dataset once again and running the method 1000 times, we varied the number of samples perturbed in the experiment. The n-fold cross-validation (one perturbed sample, in red) outperform 2-fold cross-validation (half the samples perturbed, purple). The 2-fold validation does not have an AUROC performance of 1 at 100% (~0.98), likely due to the fact that a great deal of co-expression is lost, hence preventing the method from detecting any structured co-variation. (TIF) [file pcbi.1004868.s007.tif]

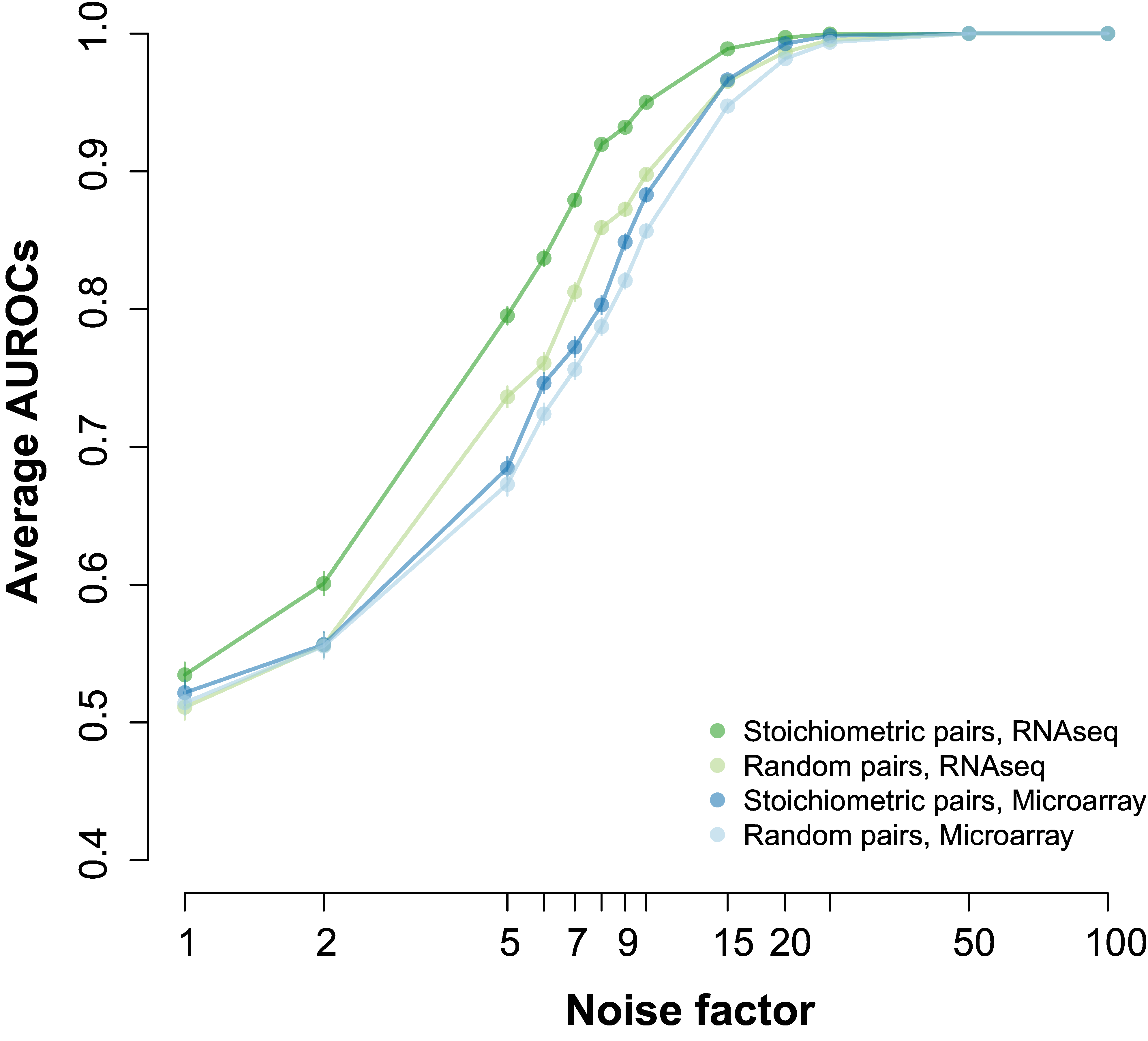

Supplement: S7 Fig — The co-expressed pairs (darker lines) outperform the random pairs (lighter lines), for both the RNA-seq (green) and Microarray (blue) datasets. But the microarray dataset has more similar performances between the co-expressed pairs and the random pairs, compared to the RNA-seq version. The microarray dataset also performs worse/close to the RNA-seq random pairs. (TIF) [file pcbi.1004868.s008.tif]

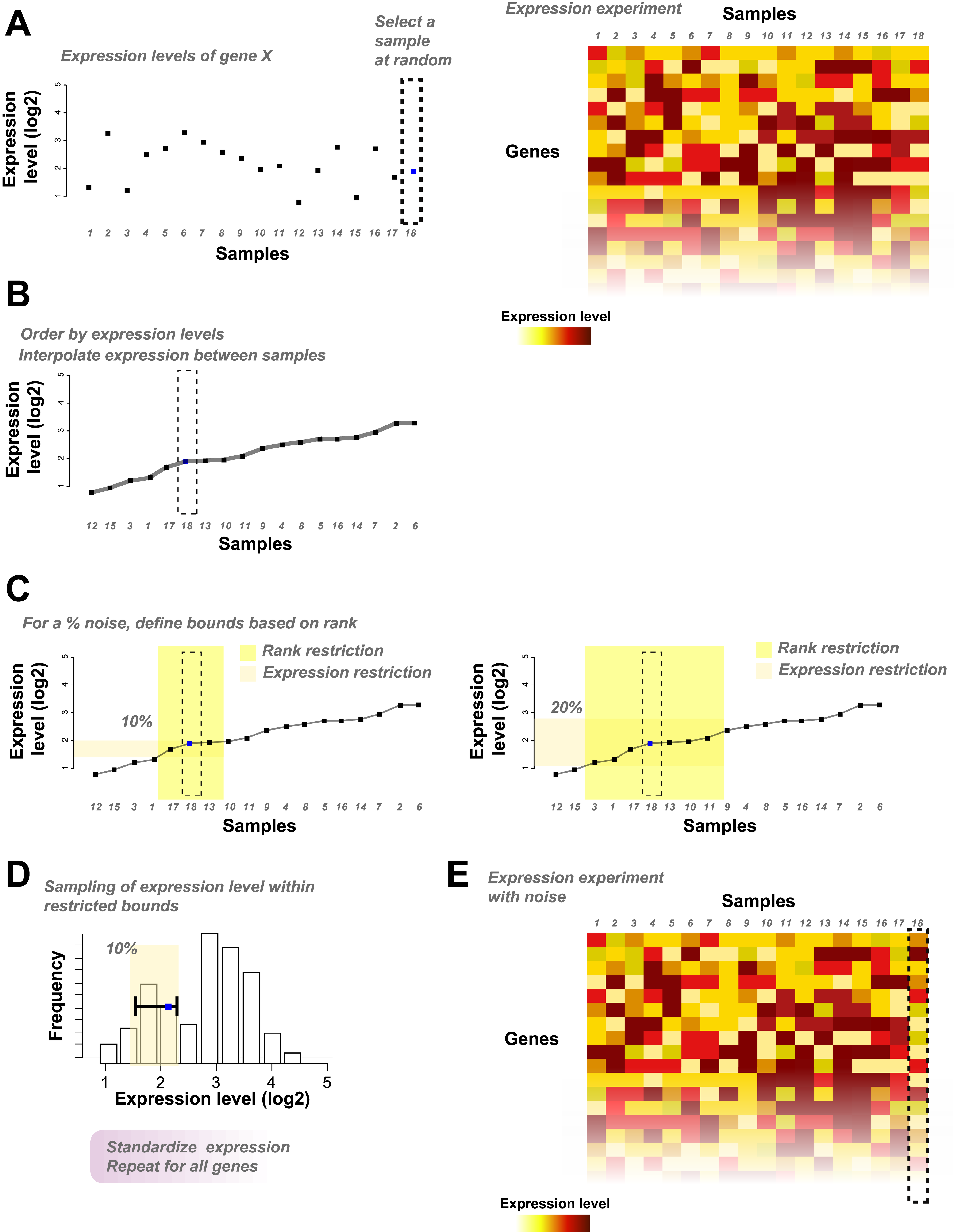

Supplement: S8 Fig — (A) Expression of gene X from experiment. (B) The first step is to order the expression levels, and interpolate between the samples to get a continuous distribution of expression. (C) Then with this distribution, we define the boundaries for the noise included expression levels for that gene. For instance, with 10% noise, the new rank is limited to the yellow rectangle bounds, which corresponds to an expression change, constrained between expression values of ~1 to ~2 (the orange rectangle). (D) We then pick the new expression level and standardize it. (E) This is repeated until all the genes in the selected sample have “noise” added to them. (TIF) [file pcbi.1004868.s009.tif]
